# Nature Inspired Computational Technique for the Numerical Solution of Nonlinear Singular Boundary Value Problems Arising in Physiology

**DOI:** 10.1155/2014/837021

**Published:** 2014-02-02

**Authors:** Suheel Abdullah Malik, Ijaz Mansoor Qureshi, Muhammad Amir, Ihsanul Haq

**Affiliations:** ^1^Department of Electronic Engineering, Faculty of Engineering and Technology, International Islamic University, Islamabad, Pakistan; ^2^Department of Electrical Engineering, Air University, Islamabad, Pakistan

## Abstract

We present a hybrid heuristic computing method for the numerical solution of nonlinear singular boundary value problems arising in physiology. The approximate solution is deduced as a linear combination of some log sigmoid basis functions. A fitness function representing the sum of the mean square error of the given nonlinear ordinary differential equation (ODE) and its boundary conditions is formulated. The optimization of the unknown adjustable parameters contained in the fitness function is performed by the hybrid heuristic computation algorithm based on genetic algorithm (GA), interior point algorithm (IPA), and active set algorithm (ASA). The efficiency and the viability of the proposed method are confirmed by solving three examples from physiology. The obtained approximate solutions are found in excellent agreement with the exact solutions as well as some conventional numerical solutions.

## 1. Introduction

The numerical treatment of nonlinear singular boundary value problems (BVPs) has been considered by several authors in the last few decades due to their varied applications in the fields of engineering and science such as gas dynamics, atomic structures, atomic calculations, and chemical reactions [1]. Many methods including finite difference method, Chebyshev polynomial, B-spline method, and nonpolynomial cubic spline have been employed to handle singular boundary value problems. The reader may find a comprehensive survey of computational techniques utilized for the numerical solution of singular boundary value problems in [1].

The key objective of this paper is to present a stochastic computing technique for the numerical solution of nonlinear singular boundary value problems of the following form [2]:

(1) $$\begin{array}{c} y^{\prime \prime }\left(x\right)+\left(a+\frac{m}{x}\right)y^{\prime }\left(x\right)=f\left(x,y\right),\;0\leq x\leq 1, \end{array}$$

(2) $$\begin{array}{c} \eta_{1}y\left(0\right)+\xi_{1}y^{\prime }\left(0\right)=\gamma_{1}, \end{array}$$

(3) $$\begin{array}{c} \eta_{2}y\left(1\right)+\xi_{2}y^{\prime }\left(1\right)=\gamma_{2}. \end{array}$$

The assumptions normally applied on *f*(*x*, *y*) are that it is continuous, ∂*f*/∂*y* exists and is continuous, and /∂*y* ≥ 0, for all 0 ≤ *x* ≤ 1. The singular boundary value problem (1)–(3) occurs in numerous applications, especially with *m* = 0, 1, 2 and *a* = 0 in the study of many tumor growth problems [3, 4] and with *m* = 2 and *a* = 0 in the study of steady state oxygen diffusion in a spherical cell with Michaelis-Menten uptake kinetics [5–8]. A similar problem for *m* = 2 and *a* = 0 also arises in the study of the distribution of heat sources in the human head [9–11].

An incredible amount of research work has been invested for the study of the singular boundary value problems (BVPs) of the form (1)–(3), and different analytical and numerical methods have been utilized [2, 12–16]. Among many authors, Rashidinia et al. employed nonpolynomial cubic spline method [2], Pandey and Singh used finite difference method [12], Khuri and Sayfy recently proposed a method based on the combination of modified decomposition method and cubic B-spline collocation [13], Çağlar et al. applied B-spline method [14], Mittal and Nigam used Adomian decomposition method (ADM) [15], and very recently Motsa and Sibanda proposed a numerical scheme based on successive linearization method (SLM) [16] for the approximate numerical solutions of singular BVPs of the form (1)–(3).

In last few decades many researchers have employed evolutionary computing based methods for handling nonlinear problems arising in engineering and science and particularly for the numerical solution of nonlinear systems of ordinary differential equations (ODEs). The efficiency and the viability of evolutionary computing (EC) based methods have been demonstrated by several authors [17–22]. Although a large number of nonlinear ODEs have been solved using the evolutionary computation based methods, only a few are narrated here. Khan et al. employed a PSO based neural network (NN) method [17] for the numerical solution Wessinger' equation. Arqub et al. used continuous genetic algorithm (CGA) based method [18] for the numerical solution of linear and nonlinear singular BVPs. Recently Malik et al. used a hybrid heuristic computing method [19] based on the combination of genetic algorithm (GA), interior point algorithm (IPA), and active set algorithm (ASA) for the numerical solution of Troesch's problem. Caetano et al. applied GA based neural network (NN) method [20] for the solution of nonlinear ODEs arising in atomic and molecular physics. Raja et al. used a hybrid PSO based neural network (NN) method [21] for the numerical solution of nonlinear Riccati differential equation of fractional order. Behrang et al. employed PSO based NN for the solution of nonlinear differential equation arising in fluid flow and heat transfer of vertical cone embedded in porous media [22].

The main focus of this work is to present an approximate numerical technique for the solution of nonlinear singular boundary value problems (1)–(3). The technique is stochastic in nature which is based on the hybrid approach of GA with two local search algorithms such as IPA and ASA. GA, IPA, ASA, and two hybrid schemes combining GA with IPA and ASA here called as GA-IPA and GA-ASA have been employed for the optimization of a fitness function which is the main thrust of the presented method. The efficiency and the viability of the presented method are demonstrated by solving three examples arising in physiology. To prove the accuracy and the validity of our results, comparisons have been carried out with the exact solutions and some conventional numerical solutions given in [2, 12–14].

The rest of the paper is arranged as follows. In Section 2 we give a brief description of the proposed methodology and heuristic search algorithms such as GA, IPA, and ASA. In Section 3 we provide numerical results that are followed by the discussion. Finally in Section 4 we give some concluding remarks and future work.

## 2. Materials and Method

### 2.1. Proposed Methodology

We may assume that the solution *y*(*x*) and its first and second derivatives *y*′(*x*) and *y*′′(*x*) can be approximated by a linear combination of some basis functions which can be represented as follows:

(4) $$\begin{array}{c} y\left(x\right)=\sum_{i=1}^{n}\alpha_{i}\varphi \left(b_{i}x+c_{i}\right), \end{array}$$

(5) $$\begin{array}{c} y^{\prime }\left(x\right)=\sum_{i=1}^{n}\alpha_{i}b_{i}\varphi^{\prime }\left(b_{i}x+c_{i}\right), \end{array}$$

(6) $$\begin{array}{c} y^{\prime \prime }\left(x\right)=\sum_{i=1}^{n}\alpha_{i}b_{i}^{2}\varphi^{\prime \prime }\left(b_{i}x+c_{i}\right), \end{array}$$

where *φ*(*x*) is taken as the log sigmoid function which is given by

(7) $$\begin{array}{c} \varphi \left(x\right)=\frac{1}{1+e^{-x}}, \end{array}$$

where *α*_*i*_, *b*_*i*_, and *c*_*i*_ are real valued unknown adjustable parameters, and *n* is the number of basis functions.

The approximate numerical solution *y*(*x*) represented by (4) is conveniently obtained once the optimum values of the adjustable parameters (*α*_*i*_, *b*_*i*_, and *c*_*i*_) are acquired. To attain the optimum values of the adjustable parameters, a problem exclusive fitness function *ε*_*j*_ is formulated. This fitness function consists of the sum of mean square error associated with the given ODE (*ε*_1_) and the mean square error related to the initial conditions (*ε*_2_), which is given as follows:

(8) $$\begin{array}{c} \varepsilon_{j}=\varepsilon_{1}+\varepsilon_{2\;}, \end{array}$$

where *j* is the cycle index and *ε*_1_ and *ε*_2_ are given by (9) and (10), respectively,

(9) $$\begin{array}{c} \varepsilon_{1}=\frac{1}{m+1}\sum_{i=0}^{m}{\left[y^{\prime \prime }\left(x_{i}\right)+\left(a+\frac{m}{x_{i}}\right)y^{\prime }\left(x_{i}\right)-f\left(x_{i},y\right)\right]}^{2}, \end{array}$$

(10) ε2=12{(η1y(0)+ζ1y′(0)−γ1)2 +(η1y(1)+ζ1y′(1)−γ2)2},

where *y*(*x*), *y*′(*x*), and *y*′′(*x*), are given by (4)–(6).

The fitness function given by (8) contains unknown adjustable parameters (*α*_*i*_, *b*_*i*_, and *c*_*i*_). The minimization of (8) is performed by utilizing the heuristic algorithms GA, IPA, and ASA, and two hybrid schemes such as GA-IPA, and GA-ASA to attain the optimal values of *α*_*i*_, *b*_*i*_, and *c*_*i*_. Consequently the approximate numerical solution of the given problem is straightforward determined from (4).

### 2.2. Brief Description of Heuristic Search Algorithms

Genetic algorithm (GA) is one of the most popular stochastic global search methods in evolutionary algorithms. GA finds the optimal solution of a problem using the evolutionary ideas of natural selection and genetics. GA starts from a randomly generated population of individuals called chromosome. Each individual within a population is regarded as a possible solution to the problem. The individuals within a population are evaluated using an exclusive fitness function of the problem at hand. The algorithm evolves population iteratively by mean three primary operations: selection, crossover, and mutation to reach the optimal solution [23].

Interior point algorithm (IPA) is a popular local search method which is widely used in varied optimization problems including linear and nonlinear, convex and nonconvex. The algorithm navigates through the interior feasible region following a central path until it reaches an optimal solution. At each iteration the algorithm applies a direct step also called Newton step or a conjugate gradient step to solve a system of Karush-Kuhn-Tucker (KKT) equations [24, 25].

Active set methods are iterative algorithms that solve a sequence of subproblems. The algorithm usually predicts and preserves a set of active and inactive constraints at an optimal solution. Generally active set methods work in two separate phases such as feasibility phase and optimality phase. In the feasibility phase the method attempts to find a feasible point for the constraints, while the objective function is ignored. In the optimality phase the method preserves the feasibility, while it attempts to find an optimal point [26].

The hybridization of GA with local search methods can provide improved performance in many optimization problems [27]. In this work we have used the hybridization of GA with two local search methods such as interior point algorithm (IPA) and active set algorithm (ASA). The GA has been used as global optimization which provides the global optimal solution which is subsequently fed into IPA and ASA for local search fine tuning to achieve the improved results.

The procedural steps of the hybrid schemes GA-IPA and GA-ASA are provided in Pseudocode 1, while the parameter settings for the implementation of these algorithms are given in Table 1.

## 3. Results and Discussion

In this section the proposed methodology is implemented on three problems arising in physiology. For the accuracy, efficacy, and viability of the proposed method, comparisons of the results are made with the exact solutions and some conventional methods including modified decomposition method (MDM) combined with B-spline collocation technique [13], B-spline functions [14], finite difference method [12], and nonpolynomial cubic spline method [2].

**Example 1**

We consider the following special case of (1) which arises in thermal explosions [13, 14]:

(11) $$\begin{array}{c} y^{\prime \prime }+\frac{1}{x}y^{\prime }={-e}^{y} \end{array}$$

subject to the boundary conditions

(12) $$\begin{array}{c} y^{\prime }\left(0\right)=0,\;\;\;y\left(1\right)=0. \end{array}$$

The exact solution of (11) is given by *y*(*x*) = 2ln⁡((*c* + 1)/(*cx* ^2^ + 1))⁡, where *c* = 3 − 2√2.

The approximate numerical solution of (11) with the given boundary conditions (12) using the proposed method is achieved by formulating its fitness function *ε*_*j*_ described in Section 2. Assuming the number of basis functions *n* = 10, the fitness function is developed as follows:

(13) $$\begin{array}{c} \varepsilon_{1}=\frac{1}{11}\sum_{i=1}^{11}{\left[y^{\prime \prime }\left(x_{i}\right)+\frac{1}{x_{i}}y^{\prime }+e^{y}\right]}^{2}, \end{array}$$

(14) $$\begin{array}{c} \varepsilon_{2}=\frac{1}{2}\left\{{\left(y^{\prime }\left(0\right)\right)}^{2}+\;{\left(y\left(1\right)\right)}^{2}\right\}, \end{array}$$

(15) $$\begin{array}{c} \varepsilon_{j}=\varepsilon_{1}+\varepsilon_{2\;}. \end{array}$$

The fitness function given by (13) is minimized by employing the algorithms GA, IPA, and ASA and two hybrid schemes GA-IPA and GA-ASA for the determination of the optimal values of unknown adjustable parameters (*α*_*i*_, *b*_*i*_, and *c*_*i*_). MATLAB 7.6 has been used for the implementation of the algorithms in this study.

The parameter settings used for the implementation of the algorithms GA, IPA, GA-IPA, and GA-ASA are given in Table 1. The length of the chromosome, that is, the number of unknown adjustable parameters (*α*_*i*_, *b*_*i*_, and *c*_*i*_), are chosen equal to 30. The values of these unknown adjustable parameters are bounded between −15 and +15. This was observed by performing many simulation experiments that, by restricting the values of unknown adjustable parameters to the interval [−15, + 15], we get better results.

The algorithms are executed according to the prescribed settings in Table 1. The optimal values of the unknown adjustable parameters corresponding to the minimum fitness are acquired. The optimal values achieved by the hybrid schemes GA-IPA and GA-ASA are given in Table 2. The optimal values of the adjustable parameters from Table 2 are used in (3) and consequently the approximate numerical solution *y*(*x*) of Example 1 is obtained. The approximate numerical results obtained by the proposed method are given in Table 3. For the accuracy of numerical results and the potency of our method, we also present the absolute errors and maximum absolute errors by our method in Tables 4 and 5, respectively. The comparisons are made with the exact solutions and the approximate numerical results obtained by other conventional methods including modified decomposition method (MDM) combined with B-spline collocation technique [13] and B-spline functions [14]. It is observed from the comparison of the absolute errors in Table 4 and the maximum absolute errors in Table 5 that the proposed method based on heuristic computing yields the approximate numerical solution of the problem given by (11)-(12) with greater accuracy. Furthermore it is evident from comparison of Table 4 that the errors relative to the exact solutions by the proposed heuristic hybrid schemes are much smaller than the errors by the approach I method used in [13], whereas they are relatively smaller than approach II method errors given in [13]. Moreover it is observed from the comparison of Table 5 that the maximum errors by the proposed method using hybrid schemes are comparable to B-spline method given in [14].

**Example 2**

We consider the following nonlinear singular boundary value problem [2]:

(16) $$\begin{array}{c} y^{\prime \prime }\left(x\right)+\left(1+\frac{m}{x}\right)y^{\prime }\left(x\right)=\frac{5x^{3}\left(5x^{5}e^{y}-x-m-4\right)}{4+x^{5}} \end{array}$$

with the boundary conditions

(17) $$\begin{array}{c} y^{\prime }\left(0\right)=0,\;\;y\left(1\right)+{5y}^{\prime }\left(1\right)=\ln\left(\frac{1}{5}\right)-5. \end{array}$$

To obtain the approximate numerical solution of (16) subject the boundary conditions (17) using the proposed method, its fitness function with *n* = 10 is developed as follows:

(18) ε1=111∑i=111[y′′(xi)+(1+mxi)y′(xi)−5xi3(5xi5ey−xi−m−4)4+xi5]2,

(19) $$\begin{array}{c} \varepsilon_{2}=\frac{1}{2}\left\{{\left(y^{\prime }\left(0\right)\right)}^{2}+{\left(\left(y\left(1\right)+{5y}^{\prime }\left(1\right)\right)-\ln\left(\frac{1}{5}\right)+5\right)}^{2}\right\}, \end{array}$$

(20) $$\begin{array}{c} \varepsilon_{j}=\varepsilon_{1}+\varepsilon_{2\;}. \end{array}$$

The heuristic algorithms GA, IPA, GA-IPA, and GA-ASA are executed with the same parameter settings given in Table 1 for the minimization of (20). To prove the effectiveness and the viability of the proposed method we have obtained the approximate numerical solutions of (16)-(17) for various values of the parameter *m* (0.25, 1, 2, and 8). The optimal values attained by the hybrid schemes corresponding to the minimum fitness are provided in Table 6 for *m* = 0.25, while for the rest of the values of *m*, these optimal values of adjustable parameters have been omitted here. For comparison maximum absolute errors have been computed corresponding to all the specified values of *m* (0.25, 1, 2, and 8). The comparison of the maximum absolute errors between the proposed heuristic method and the standard numerical methods such as finite difference method [12] and nonpolynomial cubic spline method [2] are presented in Table 7 for *m* = 0.25 and *m* = 1 and in Table 8 for *m* = 2 and *m* = 8, respectively. The comparison noticeably reveals the potency and the accuracy of the proposed method. The comparison also reveals the improved performance of hybrid schemes GA-IPA and GA-ASA.

**Example 3**

We consider Example 2 again with a change in boundary condition as follows [12]:

(21) $$\begin{array}{c} y\left(0\right)=\ln\left(\frac{1}{4}\right),\;\;y\left(1\right)+{5y}^{\prime }\left(1\right)=\ln\left(\frac{1}{5}\right)-5. \end{array}$$

The fitness function of this example is given as follows:

(22) ε1=111∑i=111[y′′(xi)+(1+mxi)y′(xi)−5xi3(5xi5ey−xi−m−4)4+xi5]2,

(23) ε2=12{(y′(0)−ln⁡(14))2 +((y(1)+5y′(1))−ln⁡(15)+5)2},

(24) $$\begin{array}{c} \varepsilon_{j}=\varepsilon_{1}+\varepsilon_{2\;}. \end{array}$$

The fitness functions given by (24) is subject to minimization using the heuristic algorithms GA, IPA, and ASA and two hybrid schemes GA-IPA and GA-ASA with the same parameter settings prescribed in Table 1 for the determination of the unknown adjustable parameters. The optimal values of the unknown adjustable parameters acquired by the hybrid schemes GA-IPA and GA-ASA are provided in Tables 9 and 10 for *m* = 0.25 and *m* = 0.75, respectively.

In Tables 11 and 12 our results are compared with the exact solutions when *m* = 0.25 and *m* = 0.75. Moreover in Table 13 we also present a comparison of the maximum absolute errors between our method and the finite difference method given in [12]. It is observed that the proposed method yields the approximate solutions fairly comparable to the finite difference method given in [12].

## 4. Conclusions and Future Work

In this study a hybrid heuristic computational approach has been successfully implemented for the approximate numerical solution of nonlinear singular boundary value problems (BVPs) arising in physiology. It can be concluded on the basis of the comparisons of the results made with the exact solutions and some of the standard approximate numerical solutions that the proposed method possesses a great potential and viability for solving nonlinear singular boundary value problems (BVPs) arising in diverse fields of engineering and science. The strength of proposed method has been illustrated by solving three nonlinear problems appearing in physiology. Moreover the proposed methodology can provide the approximate numerical solution straightforward and on a continuous grid of time once the optimal values of the unknown adjustable parameters are attained.

In future we intend to employ the proposed methodology to other such nonlinear singular boundary value problems, nonlinear ordinary differential equations (ODEs), and nonlinear coupled ODEs arising in various fields of engineering and applied science. We also seek to use other evolutionary algorithms and different basis functions for the approximate solutions of such problems.

**Pseudocode 1:**
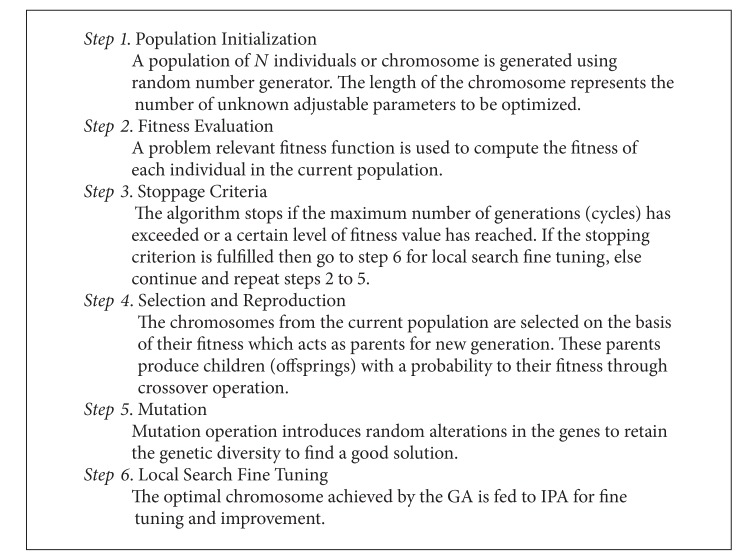
Hybridization of GA with IPA and ASA.

## Figures and Tables

**Table 1:** Parameter settings of algorithms.

GA		ASA		IPA	
Parameters	Settings	Parameters	Settings	Parameters	Settings
Population size	240	Start point	Optimal values from GA	Start point	Optimal values from GA
Chromosome size	30	Maximum iterations	400	Maximum iterations	1000
Selection function	Stochastic uniform	Maximum function evaluations	150000	Maximum function evaluations	150000
Mutation function	Adaptive feasible	Function tolerance	1*e* − 18	Function tolerance	1*e* − 18
Crossover function	Heuristic	Nonlinear constraint tolerance	1*e* − 18	Nonlinear constraint tolerance	1*e* − 18
Hybridization	PS/IPA	SQP tolerance	1*e* − 18	SQP tolerance	1*e* − 18
		X tolerance	1*e* − 18	X tolerance	1*e* − 18
Number of generations	2000			Hessian	BFGS
Function tolerance	1*e* − 18			Derivative type	Central differences
Nonlinear constraint tolerance	1*e* − 18				
Bounds	−15, +15				

**Table 2:** Optimal values of adjustable parameters acquired by hybrid schemes GA-IPA and GA-ASA for Example 1.

Algorithm	*i*	*α*_*i*_	*b*_*i*_	*c*_*i*_
GA-IPA	1	−0.881133727216563	0.175902006189556	2.693904577232850
	2	−1.914168112274790	1.024785387381440	−0.272756383809543
	3	0.901876834716753	1.555229731984290	0.208750983272920
	4	−1.947284050383540	0.246494504533957	2.816685127611610
	5	0.319195846157371	0.311493860596170	2.589042884403360
	6	0.338828303168466	1.412954189710230	2.134887086122020
	7	1.815710626779760	−1.118954968300880	2.558280772425890
	8	0.597896084699779	1.439765864844720	0.831615402493059
	9	−0.910846359413137	−2.055541748351630	−2.280854618769590
	10	0.921033436460622	−0.501229298596323	1.104201541521030

GA-ASA	1	−1.222686746438630	0.189242172392676	8.252947472264750
	2	−2.523521838558800	0.932853654818075	−1.298732098734470
	3	0.093712913166590	2.928861096270040	1.277936661162530
	4	−3.184466684228680	0.032276934636307	7.151638887800430
	5	0.841907376147177	0.624133706586784	6.016699202542360
	6	0.543579681831342	2.609855976017300	4.653625396488530
	7	2.180560092827050	−1.246422531808260	5.635726739884160
	8	1.088558307376610	1.635782026957210	0.687577406535134
	9	−1.174842130910290	−3.963774572717790	−5.326425392182380
	10	1.017060692847850	−0.833587641581821	2.237065486971310

**Table 3:** Numerical results of Example 1 by the proposed method.

*x*	Exact	GA	IPA	ASA	GA-IPA	GA-ASA
0	0.3166943676	0.3166656886	0.3167235925	0.3166903739	0.3166919976	0.3166964930
0.1	0.3132658505	0.3132354298	0.3132887292	0.3132625857	0.3132642918	0.3132672136
0.2	0.3030154228	0.3029841724	0.3030366052	0.3030123698	0.3030141051	0.3030160399
0.3	0.2860472653	0.2860174116	0.2860660073	0.2860446109	0.2860462114	0.2860481870
0.4	0.2625311275	0.2625020387	0.2625462372	0.2625289616	0.2625304715	0.2625320664
0.5	0.2326967839	0.2326670299	0.2327085884	0.2326948398	0.2326964390	0.2326970733
0.6	0.1968268057	0.1967963971	0.1968370622	0.1968247535	0.1968264983	0.1968263055
0.7	0.1552481067	0.1552187706	0.1552588254	0.1552459379	0.1552476485	0.1552472821
0.8	0.1083227634	0.1082965176	0.1083348565	0.1083208348	0.1083222537	0.1083221989
0.9	0.0564386025	0.0564160020	0.0564511385	0.0564372448	0.0564383104	0.0564384736
1.0	0.0000000000	−0.0000203826	0.0000106706	−0.0000009654	−0.0000000250	−0.0000000622

**Table 4:** Comparison of absolute errors for Example 1 between proposed method and the method given in [13].

Proposed method						MDM—cubic B-spline collocation method [13]	
*x*	GA	IPA	ASA	GA-IPA	GA-ASA	Approach I (with *N* = 20)	Approach II (with *N* = 20)
0	2.87*E* − 05	−2.92*E* − 05	2.37*E* − 06	3.99*E* − 06	−2.13*E* − 06	1.05*E* − 05	2.00*E* − 06
0.1	3.04*E* − 05	−2.29*E* − 05	1.56*E* − 06	3.26*E* − 06	−1.36*E* − 06	1.05*E* − 05	1.99*E* − 06
0.2	3.13*E* − 05	−2.12*E* − 05	1.32*E* − 06	3.05*E* − 06	−6.17*E* − 07	1.03*E* − 05	1.97*E* − 06
0.3	2.99*E* − 05	−1.87*E* − 05	1.05*E* − 06	2.65*E* − 06	−9.22*E* − 07	1.02*E* − 05	1.94*E* − 06
0.4	2.91*E* − 05	−1.51*E* − 05	6.56*E* − 07	2.17*E* − 06	−9.39*E* − 07	9.93*E* − 06	1.83*E* − 06
0.5	2.98*E* − 05	−1.18*E* − 05	3.45*E* − 07	1.94*E* − 06	−2.89*E* − 07	9.62*E* − 06	1.78*E* − 06
0.6	3.04*E* − 05	−1.03*E* − 05	3.07*E* − 07	2.05*E* − 06	5.00*E* − 07	6.93*E* − 06	1.67*E* − 06
0.7	2.93*E* − 05	−1.07*E* − 05	4.58*E* − 07	2.17*E* − 06	8.25*E* − 07	4.75*E* − 06	1.34*E* − 06
0.8	2.62*E* − 05	−1.21*E* − 05	5.10*E* − 07	1.93*E* − 06	5.64*E* − 07	2.93*E* − 06	9.20*E* − 07
0.9	2.26*E* − 05	−1.25*E* − 05	2.92*E* − 07	1.36*E* − 06	1.29*E* − 07	1.37*E* − 06	4.57*E* − 07
1.0	2.04*E* − 05	−1.07*E* − 05	2.50*E* − 08	9.65*E* − 07	6.22*E* − 08	0	0

**Table 5:** Comparison of maximum absolute error for Example 1 between the proposed method and the methods given in [13, 14].

Proposed method	Method in [13] Approach I	Method in [13] Approach II	B-spline method [14]
3.13*E* − 5 ( GA)	1.05*E* − 5 (*N* = 5)	3.22*E* − 5 (*N* = 5)	3.16*E* − 5 (*h* = 1/20)
2.92*E* − 5 (IPA)	1.05*E* − 5 (*N* = 10)	8.06*E* − 6 (*N* = 10)	7.87*E* − 6 (*h* = 1/40)
2.37*E* − 6 (ASA)	1.05*E* − 5 (*N* = 20)	2.00*E* − 6 (*N* = 20)	3.50*E* − 6 (*h* = 1/60)
3.99*E* − 6 (GA-IPA)			1.55*E* − 6 (*h* = 1/90)
2.13*E* − 6 (GA-ASA)			4.97*E* − 7 (*h* = 1/161)

**Table 6:** Optimal values of adjustable parameters acquired by the hybrid schemes GA-IPA and GA-ASA (for *m* = 0.25).

Algorithm	*i*	*α*_*i*_	*b*_*i*_	*c*_*i*_
GA-IPA	1	2.00000990197459	−2.53327380955379	−2.04436628299064
	2	−1.28602583694444	0.628600926323132	−3.08615267144563
	3	0.765196864102529	−0.826071194697222	−0.448002389749568
	4	−3.36316154283773	1.94124108704907	−2.24922902674485
	5	−2.81754759022593	1.90734595147276	−2.66329404646048
	6	−0.749086831887256	−3.10918782644835	1.59601601647687
	7	−2.56750443242766	−1.64680511116928	−2.3695938077251
	8	1.67102584035366	3.3098175417873	−2.95032975955236
	9	−2.91926666202298	−1.4378519528881	−1.43522291979122
	10	−0.516212440164525	−0.623509775567634	−2.703478256228

GA-ASA	1	0.464500439684582	−11.0028781120949	−12.7636935584212
	2	−3.33324694261012	2.11315018577675	−5.37188638435383
	3	−0.194337559721311	0.47470727444725	−0.042351193375573
	4	−3.45408759766678	2.70099441007266	−3.75724365940641
	5	−5.48801698503117	−14.9999432116995	−13.806030007752
	6	−1.28645991543674	−2.97502002092667	2.6266679780155
	7	−6.56302549714287	−5.15963787587387	−8.09010590696137
	8	1.9884484940218	2.42668650849131	−5.97859767484604
	9	−9.05563942802748	−1.15701411492891	−11.8680893657959
	10	−1.05136593168004	−1.23760873949133	−10.0917104751297

**Table 7:** Comparison of maximum absolute errors in solution of Example 2 between the proposed heuristic computing method and the methods given in [2, 12] (for *m* = 0.25, 1).

*m* = 0.25			*m* = 1		
Proposed method	Method in [12]	Method in [2]	Proposed method	Method in [2]	Method in [12]
1.11*E* − 4 (GA)	1.17*E* − 4 (*N* = 16)	2.07*E* − 4 (*N* = 16)	6.46*E* − 4 (GA)	1.46*E* − 3 (*N* = 16)	1.71*E* − 3 (*N* = 16)
1.10*E* − 4 (IPA)	3.04*E* − 4 (*N* = 32)	1.87*E* − 4 (*N* = 32)	1.43*E* − 4 (IPA)	3.68*E* − 4 (*N* = 32)	1.87*E* − 4 (*N* = 32)
1.42*E* − 4 (ASA)	7.67*E* − 5 (*N* = 64)	3.88*E* − 5 (*N* = 64)	3.23*E* − 4 (ASA)	9.20*E* − 5 (*N* = 64)	1.96*E* − 5 (*N* = 64)
6.47*E* − 5 (GA-IPA)	1.92*E* − 5 (*N* = 128)	8.10*E* − 5 (*N* = 128)	1.14*E* − 5 (GA-IPA)	2.30*E* − 5 (*N* = 128)	1.72*E* − 5 (*N* = 128)
1.40*E* − 4 (GA-ASA)	4.81*E* − 6 (*N* = 256)	2.75*E* − 6 (*N* = 256)	1.51*E* − 4 (GA-ASA)	5.75*E* − 6 (*N* = 256)	1.77*E* − 6 (*N* = 256)

**Table 8:** Comparison of maximum absolute errors in solution of Example 2 between the proposed heuristic computing method and the methods given in [2, 12] (for *m* = 2, 8).

*m* = 2			*m* = 8	
Proposed method	Method in [12]	Method in [2]	Proposed method	Method in [2]
9.04*E* − 2 (GA)	1.82*E* − 3 (*N* = 16)	7.71*E* − 3 (*N* = 16)	1.11*E* − 4 (GA)	4.11*E* − 3 (*N* = 16)
1.26*E* − 4 (IPA)	4.52*E* − 4 (*N* = 32)	7.78*E* − 5 (*N* = 32)	1.1*E* − 4 (IPA)	9.76*E* − 4 (*N* = 32)
9.52*E* − 5 (ASA)	9.20*E* − 5 (*N* = 64)	7.05*E* − 5 (*N* = 64)	1.42*E* − 4 (ASA)	2.38*E* − 4 (*N* = 64)
4.29*E* − 4 (GA-IPA)	2.80*E* − 5 (*N* = 128)	6.45*E* − 6 (*N* = 128)	6.47*E* − 5 (GA-IPA)	5.89*E* − 5 (*N* = 256)
5.47*E* − 5 (GA-ASA)	7.00*E* − 6 (*N* = 256)	7.38*E* − 7 (*N* = 256)	1.40*E* − 4 (GA-ASA)	3.66*E* − 6 (*N* = 512)

**Table 9:** Optimal values of adjustable parameters acquired by the hybrid schemes GA-IPA and GA-ASA (for *m* = 0.25).

Algorithm	*i*	*α*_*i*_	*b*_*i*_	*c*_*i*_
GA-IPA	1	−1.21283109847133	2.10611144213430	−1.74180188323508
	2	1.03571174166241	3.33901417043102	1.97927968097166
	3	2.54354943860472	−2.81356919290601	−1.82477617426169
	4	−0.08380302331005	−0.89090920684382	−3.47294702330385
	5	−3.78578617501133	2.34830432992743	−0.46468044922713
	6	0.77113283421175	1.39103539510805	0.60221633923140
	7	−0.39166723764742	−2.78667344812987	−2.71312485832199
	8	2.60276365782489	3.24107191442605	2.25289685558876
	9	−4.01442973095422	0.11578635663362	−1.55798487398090
	10	−0.01034412572293	−0.73422658403828	−1.74180188323508

GA-ASA	1	−1.29954899127975	1.55618946670852	−4.97951286549580
	2	0.36030995762761	4.54268965985819	−1.53639071233373
	3	1.35509804608392	−3.67898087578096	1.22063364702299
	4	−1.49517350846173	−8.31269717983695	−6.46006519243648
	5	−3.56649523601647	2.84611416572567	−4.07363209952837
	6	0.13571937537889	1.49401185018163	−1.64402410793090
	7	−1.17448382336998	2.18102189143932	1.16234155281522
	8	3.14946103860480	1.98119612209956	−0.89293215000584
	9	−2.50385665347528	1.39658982973737	4.20709618704552
	10	−0.21700379010878	−2.35736445901048	−5.76189602761190

**Table 10:** Optimal values of adjustable parameters acquired by the hybrid schemes GA-IPA and GA-ASA (for *m* = 0.75).

Algorithm	*i*	*α*_*i*_	*b*_*i*_	*c*_*i*_
GA-IPA	1	−2.83258569000288	2.98033056807418	−5.23172928318637
	2	−4.42010332472099	0.13495932817614	4.02502515752573
	3	3.30600805415091	2.08467745171526	4.03825862924909
	4	0.00408821799800	8.27613404808222	1.33095013889441
	5	1.32492260599249	−3.71698529742389	4.55970043938072
	6	−2.30918802461094	4.73975316526285	6.29762522267394
	7	1.71075781492918	2.37576486096926	−2.55486267649637
	8	4.52199590675746	−4.38217408351950	−7.46847005821201
	9	0.28578832784628	−1.39979371265597	2.03514844007566
	10	0.55554404620260	−1.63197937784219	0.41337265836928

GA-ASA	1	−2.73056565691944	3.49085467009396	−5.25776847865554
	2	−4.33634711954231	0.23184895540015	4.31738960575493
	3	3.80423016883327	1.80591217864829	4.67576012584760
	4	0.01364375729467	9.41077798745202	1.15634974437215
	5	0.80980144878077	−3.95901379093358	4.45931462422107
	6	−2.33747274025478	5.28336935304114	6.13742438292330
	7	1.88177593528183	2.61799608130219	−3.08482698001940
	8	4.88126374826236	−4.73664684075954	−8.02792576795810
	9	0.40997080411581	−1.47729741688129	2.21958697592547
	10	0.33789053645730	−1.33529803313717	0.44393611859182

**Table 11:** Approximate numerical results of Example 3 (for *m* = 0.25) by the proposed method.

*x*	Exact	GA	IPA	ASA	GA-IPA	GA-ASA
0.1	−1.386296861	−1.38624919	−1.386296338	−1.386262628	−1.386296203	−1.386298753
0.2	−1.386374358	−1.386352825	−1.386374473	−1.386378504	−1.386376846	−1.386391479
0.3	−1.386901677	−1.386878034	−1.386901875	−1.386941133	−1.386906884	−1.386932751
0.4	−1.388851090	−1.388860232	−1.388851841	−1.388925666	−1.388858277	−1.388889837
0.5	−1.394076502	−1.394112267	−1.394077442	−1.394174061	−1.394085067	−1.394124318
0.6	−1.405547818	−1.405577315	−1.405548911	−1.405653799	−1.405558190	−1.405605243
0.7	−1.427453099	−1.42749144	−1.427454577	−1.427568614	−1.427464669	−1.427513251
0.8	−1.465031602	−1.465118065	−1.465033212	−1.465167671	−1.465043706	−1.465096028
0.9	−1.523986772	−1.524106136	−1.523988676	−1.524137607	−1.524000240	−1.52405989
1.0	−1.609437912	−1.609549628	−1.609439991	−1.609586337	−1.609451732	−1.609511922

**Table 12:** Approximate numerical results of Example 3 (for *m* = 0.75) by the proposed method.

*x*	Exact	GA	IPA	ASA	GA-IPA	GA-ASA
0.1	−1.386296861	−1.386242375	−1.3862993910	−1.386375450	−1.386296290	−1.3863373760
0.2	−1.386374358	−1.387183465	−1.3863751180	−1.386502926	−1.386372340	−1.3864155390
0.3	−1.386901677	−1.388226417	−1.3869011670	−1.387062656	−1.386898891	−1.3869443860
0.4	−1.388851090	−1.390515300	−1.3888500510	−1.389039265	−1.388847721	−1.3888928150
0.5	−1.394076502	−1.395971989	−1.3940748480	−1.394277913	−1.394072825	−1.3941203720
0.6	−1.405547818	−1.407612573	−1.4055457830	−1.405749932	−1.405543725	−1.4055940940
0.7	−1.427453099	−1.429641077	−1.4274510240	−1.427661399	−1.427448708	−1.4274983690
0.8	−1.465031602	−1.467294836	−1.4650293580	−1.465258995	−1.465027141	−1.4650765090
0.9	−1.523986772	−1.526277928	−1.5239846410	−1.524225647	−1.523982007	−1.5240343800
1.0	−1.609437912	−1.611726339	−1.6094358900	−1.609670616	−1.609433051	−1.6094855960

**Table 13:** Comparison of maximum absolute errors for Example 3 between the proposed method and the method given in [12].

*m* = 0.25		*m* = 0.75	
Proposed method	Finite difference method [12]	Proposed method	Finite difference method [12]
1.11*E* − 4 (GA)	7.85*E* − 4 (*N* = 16)	6.46*E* − 4 (GA)	7.94*E* − 4 (*N* = 16)
1.10*E* − 4 (IPA)	1.94*E* − 4 (*N* = 32)	1.43*E* − 4 (IPA)	2.00*E* − 4 (*N* = 32)
1.42*E* − 4 (ASA)	4.83*E* − 5 (*N* = 64)	3.23*E* − 4 (ASA)	5.00*E* − 5 (*N* = 64)
6.47*E* − 5 (GA-IPA)	1.21*E* − 5 (*N* = 128)	1.14*E* − 5 (GA-IPA)	1.25*E* − 5 (*N* = 128)
1.40*E* − 4 (GA-ASA)	3.01*E* − 6 (*N* = 256)	1.51*E* − 4 (GA-ASA)	3.13*E* − 6 (*N* = 256)
